# The relationships between LncRNA NNT-AS1, CRP, PCT and their interactions and the refractory mycoplasma pneumoniae pneumonia in children

**DOI:** 10.1038/s41598-021-81853-w

**Published:** 2021-01-21

**Authors:** Ping Chen, Zheng Huang, Lumin Chen, Shihao Zhuang, Hongli Lin, Jianfeng Xie, Kuicheng Zheng

**Affiliations:** 1 Fujian Children’s Hospital, Fujian, China; 2 Fujian Center for Disease Control and Prevention, Fujian, China; 3 Fujian Maternity and Child Health Hospital, Fujian, China

**Keywords:** RNA, Biomarkers, Diseases, Risk factors

## Abstract

To investigate the relationships between LncRNA NNT-AS1, CRP, PCT and their interactions and the refractory mycoplasma pneumoniae pneumonia (RMPP) in children. Serum levels of LncRNA NNT-AS1 of RMPP and non-RMPP (NRMPP) patients were detected by real-time PCR, and were analyzed together with serum c-reactive protein (CRP) and procalcitonin (PCT). Correlations between LncRNA NNT-AS1 and CRP and PCT were analyzed by Pearson correlation test. The ROC curve was used to analyze the potential of LncRNA NNT-AS1, CRP and PCT as biomarkers for predicting RMPP. Logistic regression crossover model and the Excel compiled by Andersson et al. were used to analyze the interactions among the biomarkers. We found that LncRNA NNT-AS1, CRP and PCT were all highly expressed in patients with RMPP. LncRNA NNT-AS1 could positively correlate with the expressions of CRP and PCT, and jointly promote the occurrence of RMPP. The combined diagnosis of LncRNA NNT-AS1, CRP and PCT could predict the occurrence of RMPP.

## Introduction

Mycoplasma pneumoniae (MP) has become one of the common pathogens of children's respiratory tract infection, especially in community-acquired pneumonia^1^. Mycoplasma pneumoniae pneumonia (MPP) is more commonly seen in school-age children and adolescents, and even reaches 70% among people with a high prevalence^2^. Most MPP patients have mild clinical symptoms and could recover quickly after treated with macrolides antibiotics or when their immunity had improved, yet there are still refractory mycoplasma pneumoniae pneumonia (RMPP) patients who are not responsive to macrolides antibiotic treatments, some may develop atelectasis, occlusive bronchiolitis, pleural effusion, and/or necrotizing pneumonia, and become critically ill or even death^3^. In China, the resistance rate of MP to macrolides is as high as 83–95%^4,5^.

C-reactive protein (CRP), an acute time phase protein that increases when body has inflammatory responses and tissue damages caused by infections, is an important indicator in diagnosing pneumonia in children^6^. Procalcitonin (PCT), a 116-amino-acid glycoprotein secreted by thyroid C cells and exists in free form in the serum, is a sensitive indicator for infections^7^. Serum PCT is abnormally elevated and positively correlated with the severity of the conditions in patients with bacterial infections, burns, pancreatitis, and multiple trauma^8^. Studies also showed that serum CRP and PCT levels had certain predictive values for the prognosis of hospitalized community-acquired pneumonia^9^.

Long noncoding RNA (lncRNA) is a class of non-coding sequences with a length of more than 200 nucleotides. Due to the lack of open reading framework, lncRNA is not capable of coding proteins^10^. Studies have shown that the frequent abnormal expressions of lncRNA in tissues and cells may be closely related to the occurrence and development, invasion and metastasis of tumors and drug resistance^11^. Gu et al.^12^ found that LncRNA MALAT1 expression was higher in children with MPP.In addition, studies have found that LncRNA ZFAS1 Promotes Acute Myocardial Infarction by Regulating CRP^13^. LncRNA NNT-AS1, located on human chromosome 5, is a newly discovered LncRNA. Wang et al.^14^ found in combination with in vitro and in vivo experiments that LncRNA NNT-AS1 was highly expressed in colorectal cancer tissues and could promote the proliferation, metastasis and invasion of colorectal cancer cells by regulating the MAPK/ERK signaling pathway and the epithelial-mesenchymal transformation of tumor cells. Other research has shown that LncRNA NNT-AS1 was relevant to the drug resistance of non-small cell lung cancer (NSCLC), Cai Y et al.^15^ found that LncRNA NNT-AS1 were higher in the drug-resistant cells and the drug-resistance tissues of NSCLC than in the parental cells and the paracancerous tissues, and that interference with the LncRNA NNT-AS1 expression could inhibit the proliferation of the drug-resistant cells and promote apoptosis and cell cycle arrest, indicating that LncRNA NNT-AS1 could promote cisplatin resistance in NSCLC cells. However, there has been no study on the correlation between RMPP and the expressions of lncRNA NNT-AS1, CRP and PCT. Therefore, in this study, the expressions of lncRNA NNT-AS1, CRP and PCT were detected and their correlations with RMPP were analyzed to achieve early diagnosis and treatment, thus shortening the course of the disease and reducing the occurrence of related sequelae.

## Results

### Comparisons on the demographic and clinical characteristics between NRMPP and RMPP

A total of 150 subjects were collected in this study, including 83 NRMPP children with a male/female ratio of 52/31 and an average age of (4.31 ± 2.06) years old, and 67 RMPP children with a male/female ratio of 39/28 and an average age of (4.73 ± 2.65) years old. Statistical analysis results showed that there was no statistical differences in gender and age between the children with NRMPP and RMPP in this study (P > 0.05). Compared with NRMPP cases, fever time and hospital stay were longer in RMPP cases (both P < 0.05). However, the incidence of abnormal pulmonary signs in NRMPP cases was higher than that in RMPP cases (P < 0.05) (Table 1). In addition, we compared the blood tests of all our subjects, which showed that the IgA and IgG levels in children with RMPP were significantly higher than those in children with NRMPP (P < 0.05) (Table 2). In all, the NRMPP and RMPP subjects in this study were different in fever time, length of hospital stay, incidence of abnormal pulmonary signs, and IgA and IgG expressions.

**Table 1:** Comparison of demographic data and clinical characteristics between NRMPP and RMPP.

Variable	NRMPP (n = 83)	RMPP (n = 67)	*t/χ*^*2*^	*P*
Sex (male/female)	52/31	39/28	0.307	0.617
Age (years old)	4.31 ± 2.06	4.73 ± 2.65	− 1.092	0.277
**Clinical profiles**				
Fever (days)	3.05 ± 3.21	7.14 ± 4.66	− 6.347	< 0.001
Hospital stay (days)	7.17 ± 6.32	12.98 ± 9.34	− 4.528	< 0.001
Cough (%)	75 (90.36%)	58 (86.57%)	0.531	0.606
Nose running (%)	32 (38.55%)	20 (29.85%)	1.240	0.303
Swelling (%)	44 (53.01%)	43 (64.18%)	1.898	0.186
Abnormal signs of lungs (%)	51 (61.45%)	23 (34.33%)	10.907	0.001
Diarrhea (%)	3 (3.61%)	1 (1.49%)	0.643	0.629

**Table 2:** Comparison of clinical and biochemical between NRMPP and RMPP Patients.

Variable	NRMPP (n = 83)	RMPP (n = 67)	*T*	*P*
WBC (× 10^9^/L)	8.16 ± 3.45	7.87 ± 2.55	0.573	0.567
Neutrophils (× 10^9^/L)	4.73 ± 2.22	5.17 ± 2.63	− 1.111	0.268
Platelets (× 10^9^/L)	316.77 ± 94.42	321.84 ± 94.75	− 0.326	0.745
IgA (g/L)	0.74 ± 0.37	1.13 ± 0.52	− 5.358	< 0.001
IgG (g/L)	7.93 ± 2.83	10.63 ± 3.08	− 5.584	< 0.001
IgM (g/L)	1.49 ± 0.75	1.62 ± 1.05	− 0.883	0.379
LDH (U/L)	292.14 ± 58.84	294.68 ± 59.41	− 0.262	0.794
ESR (mm/h)	30.53 ± 7.96	30.04 ± 8.47	0.364	0.716
d-Dimer (μg/L)	716.03 ± 127.58	686.85 ± 93.59	1.563	0.120

*WBC* white blood cell count, *LDH* lactate dehydrogenase, *ESR* erythrocyte sedimentation rate.

### Serum expressions of LncRNA NNT-AS1, CRP and PCT between NRMPP and RMPP

**Figure 1:**
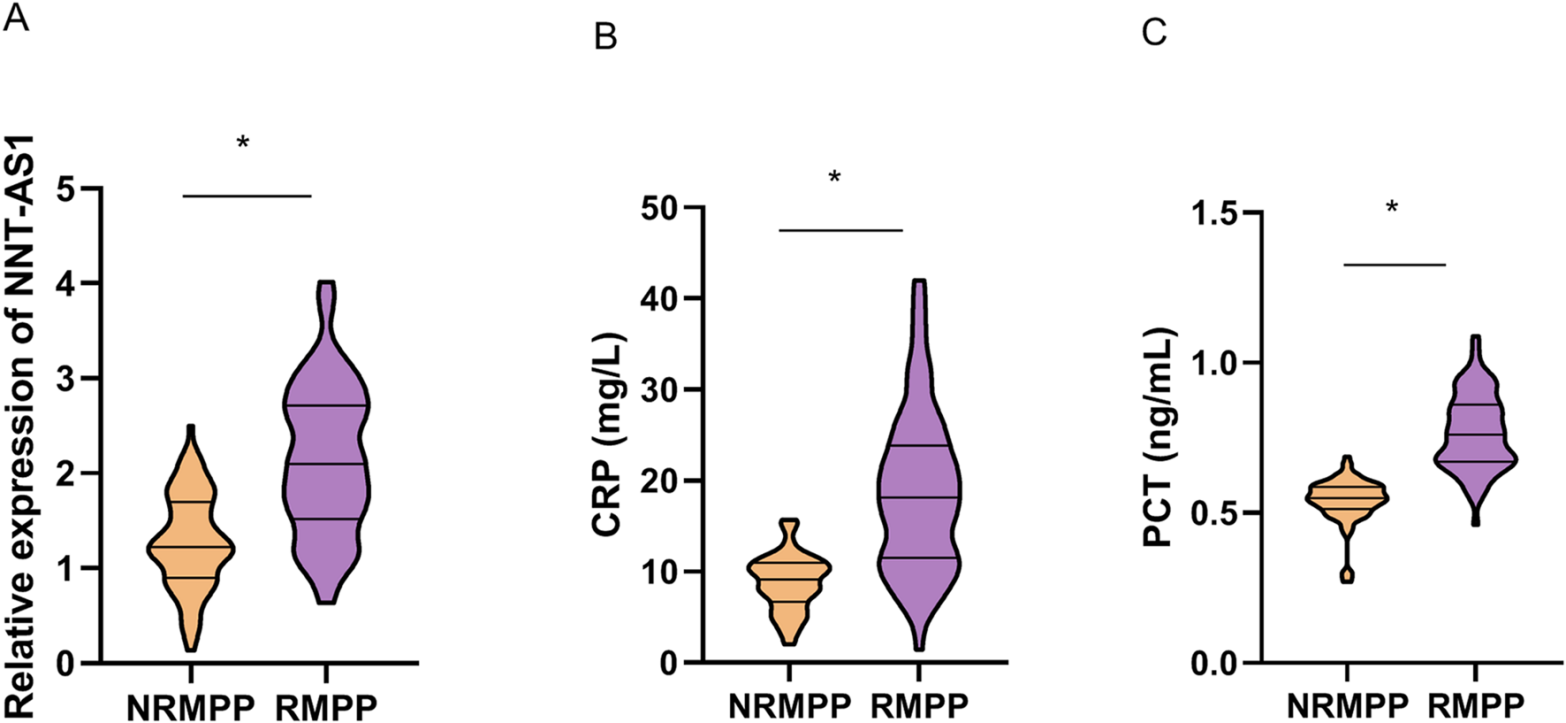
Serum expressions of LncRNA NNT-AS1, CRP and PCT between NRMPP and RMPP. **(A)** Detection by qRT-PCR, the serum expression of LncRNA NNT-AS1 in RMPP children was significantly higher than that in NRMPP children. **(B)** Blood test results showed that compared with NRMPP children, serum expression of CRP was significantly increased in RMPP children. **(C)** Blood test results showed that compared with NRMPP children, serum expression of PCT was significantly increased in RMPP children.

Results from qRT-PCR showed that the serum expressions of LncRNA NNT-AS1 in the 67 RMPP children were significantly higher than that in NRMPP children (P < 0.05) (Fig. 1A). Blood test results showed that compared with NRMPP children, serum CRP (Fig. 1B) and PCT (Fig. 1C) were also significantly increased in RMPP children (both P < 0.05). These results indicated that the abnormal expressions of LncRNA NNT-AS1, CRP and PCT may be correlated with the occurrence and development of RMPP.

### Correlations between LncRNA NNT-AS1 and relevant clinical indicators

Results showed that IgA, IgG, CRP and PCT may affect RMPP development. In order to further explore whether the expressions of IgA, IgG, CRP and PCT levels are related with LncRNA NNT-AS1, the 150 objects were divided into high expression and low expression groups, each with 75 objects. Statistical results showed that, the expression levels of CRP and PCT in the high expression LncRNA NNT-AS1 group were higher than those in the low expression group, with statistically significant difference (P < 0.05), while the expression levels of IgA and IgG in the two groups were not statistically significant different (P > 0.05) (Table 3). These results suggested that the expressions of CRP and PCT in serum of RMPP patients may be related to LncRNA NNT-AS1.

**Table 3:** Correlation between clinical laboratory indicators and LncRNA NNT-AS1.

Variable	High level of NNT-AS1 (n = 75)	Low level of NNT-AS1 (n = 75)	*t*	*P*
IgA (g/L)	0.92 ± 0.33	0.97 ± 0.41	− 0.823	0.412
IgG (g/L)	9.98 ± 2.71	9.51 ± 2.63	1.078	0.283
CRP (mg/L)	38.54 ± 9.42	10.82 ± 5.23	22.281	< 0.001
PCT (ng/mL)	1.72 ± 0.42	0.93 ± 0.26	13.850	< 0.001

### Correlations among LncRNA NNT-AS1, CRP and PCT

**Figure 2:**
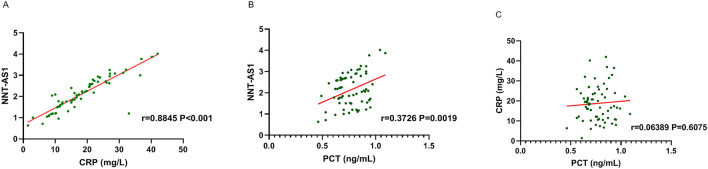
Correlations among LncRNA NNT-AS1, CRP and PCT. **(A)** Pearson correlation test showed that serum LncRNA NNT-AS1 was positively correlated with CRP expression (r = 0.8845, P < 0.001). **(B)** Pearson correlation test showed that serum LncRNA NNT-AS1 was positively correlated with PCT expression (r = 0.3726, P = 0.0019). C. Pearson correlation test showed that there was no linear correlation between CRP and PCT expression (r = 0.06389, P = 0.6075).

To further clarify the correlations between CRP and PCT expressions and LncRNA NNT-AS1,we used Pearson correlation test to analyze. Results showed that LncRNA NNT-AS1 was positively correlated with both CRP (r = 0.8845, P < 0.001) (Fig. 2A) and PCT (r = 0.3726, P = 0.0019) (Fig. 2B) in RMPP children. However no linear correlation was found between CRP and PCT (r = 0.06389, P = 0.6075) (Fig. 2C). These results indicated that LncRNA NNT-AS1 was positively correlated with CRP and PCT.

### Predictability of LncRNA NNT-AS1, CRP and PCT on RMPP

**Figure 3:**
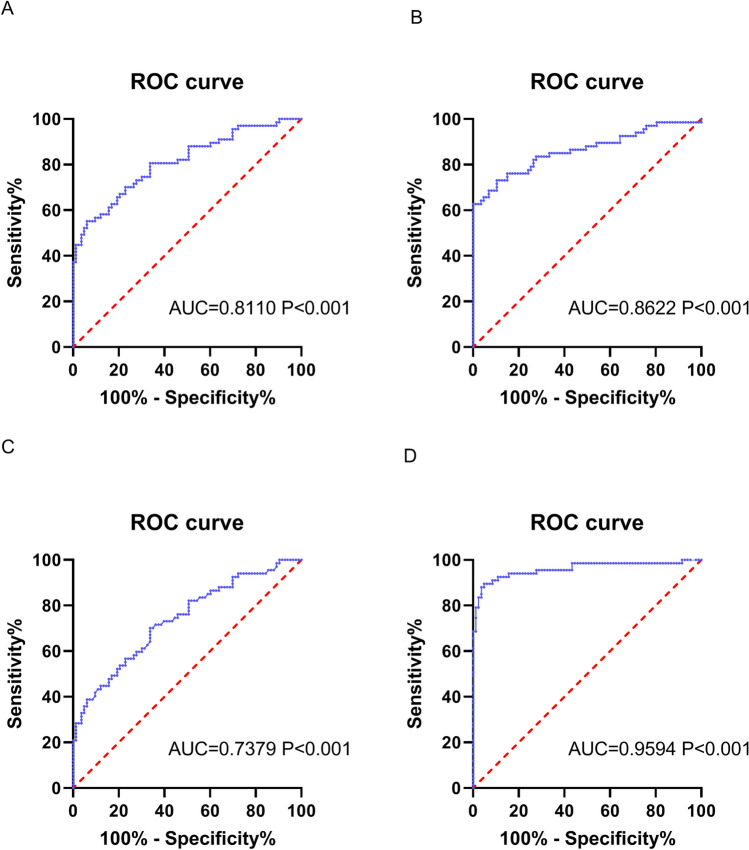
Predictability of LncRNA NNT-AS1, CRP and PCT on RMPP. **(A)** ROC curve analyzed that the AUC of LncRNA NNT-AS1 prediction on RMPP was 0.8110. **(B)** ROC curve analyzed that the AUC of CRP prediction on RMPP was 0.8622. **(C)** ROC curve analyzed that the AUC of PCT prediction on RMPP was 0.7379. **(D)** ROC curve analyzed that the AUC of the combined prediction of LncRNA NNT-AS1, CRP and PCT on RMPP was 0.9594 (P < 0.001).

To further explore whether LncRNA NNT-AS1, CRP and PCT could be potential biomarkers for predicting RMPP, the ROC curve analysis were performed and the results showed that the AUCs of LncRNA NNT-AS1, CRP and PCT were 0.8110, 0.8622 and 0.7379, respectively (all P < 0.05, Fig. 3A–C). The AUC of the joint diagnosis of the three parameters on RMPP was 0.9594 (P < 0.001, Fig. 3D), with the cut-off value that had obtained the maximum sensitivities and specificities in the separate predictions of LncRNA NNT-AS1, CRP and PCT. These results indicated that the combined diagnosis of LncRNA NTN-AS1, CRP and PCT could very well predict the occurrence of RMPP.

### Logistic regression analysis of the effects of LncRNA NNT-AS1, CRP and PCT on RMPP

In order to explore whether LncRNA NNT-AS1, CRP and PCT might be risk factors for RMPP, the RMPP subjects in this study were divided into different groups of high and low LncRNA NNT-AS1, CRP and PCT, respectively, using their respective cut-off values as the separation limits. Univariate Logistic regression analysis showed that LncRNA NNT-AS1, CRP and PCT were all risk factors for RMPP. After adjusting for confounding factors (model 3), the OR(95%CI) of LncRNA NNT-AS1, CRP and PCT were 1.547 (1.434–2.973), 1.472 (1.273–2.084) and 1.786 (1.573–2.148), respectively (Table 4). These results indicated that the increased expressions of LncRNA NT-AS1, CRP and PCT could increase the risk of RMPP.

**Table 4:** Logistic analysis of risk factors for RMPP.

Variable	Model I			Model II			Model III	
	OR (95%CI)	P		OR (95%CI)	P		OR (95%CI)	P
High level of CRP	1.738 (1.219–3.883)	0.004		1.764 (1.489–3.167)	0.002		1.547 (1.434–2.973)	0.003
High level of PCT	1.526 (1.284–2.462)	0.027		1.512 (1.144–2.218)	0.015		1.472 (1.273–2.084)	0.009
High level of LncRNA NNT-AS1	2.024 (1.725–3.746)	< 0.001		1.863 (1.742–2.642)	< 0.001		1.786 (1.573–2.148)	< 0.001

Model II: adjusting the fever days, hospital stay days, abnormal signs of lungs on the basis of Model I; Model III: adjusting IgA, IgG on the basis of Model II.

### Interactions among LncRNA NNT-AS1, CRP and PCT

After adjusting for confounding factors, it was found that in patients with both high LncRNA NNT-AS1 and CRP, the risk of RMPP was 3.281 times than that in patients with both low measurements (95%CI: 1.673–3.651); and the risk of RMPP in patients with both high LncRNA NNT-AS1 and PCT was 1.983 times (95%CI: 1.486–3.571) than that in patients with both low measurements (Table 5). After adjusting for confounding factors, the effects of the high LncRNA NNT-AS1 and CRP pair and the high LncRNA NNT-AS1 and PCT pair were 1.531 times and 1.186 times, respectively, than the sums of the effects of the unpaired factors (S). The RMPP explained by the interactions of this two pairs, respectively, were 2.753 times and 1.164 times (RERI) than the RMPP explained by other unmeasured factors. Of the RMPP risks in the high LncRNA NNT-AS1 and CRP pair and in the high LncRNA NT-AS1 and PCT pair, there were 41.5% and 32.4% (AP%), respectively, caused by the interactions; However there was no additive interaction found between CRP and PCT (Table 6). These results showed that LncRNA NNT-AS1 interacted with CRP and PCT by multiplication and positive addition.

**Table 5:** Multiplicative interaction of related indicators.

Variable		Model I		Model II		Model III	
		OR (95% CI)	*P*	OR (95% CI)	*P*	OR (95% CI)	*P*
**NNT-AS1 & CRP**							
Low level of NNT-AS1	Low level of CRP	1		1		1	
Low level of NNT-AS1	High level of CRP	1.634 (1.231–2.144)	< 0.001	1.693 (1.374–2.317)	< 0.001	1.541 (1.203–1.986)	< 0.001
High level of NNT-AS1	Low level of CRP	1.973 (1.367–2.725)	< 0.001	1.998 (1.504–2.864)	< 0.001	1.783 (1.226–2.184)	< 0.001
High level of NNT-AS1*High level of CRP		3.205 (1.779–4.332)	< 0.001	3.375 (1.853–4.376)	< 0.001	3.281 (1.673–3.651)	< 0.001
**NNT-AS1 & PCT**							
Low level of NNT-AS1	Low level of PCT	1		1		1	
Low level of NNT-AS1	High level of PCT	1.325 (1.254–2.364)	0.028	1.485 (1.284–2.641)	0.021	1.277 (1.214–2.071)	0.018
High level of NNT-AS1	Low level of PCT	1.754 (1.315–2.551)	< 0.001	1.818 (1.441–2.604)	< 0.001	1.654 (1.241–2.314)	< 0.001
High level of NNT-AS1*High level of PCT		2.145 (1.631–3.743)	< 0.001	2.284 (1.752–3.788)	< 0.001	1.983 (1.486–3.571)	< 0.001
**CRP & PCT**							
Low level of CRP	Low level of PCT	1		1		1	
Low level of CRP	High level of PCT	1.365 (1.164–2.846)	0.037	1.487 (1.289–2.978)	0.025	1.317 (1.145–2.285)	0.022
High level of CRP	Low level of PCT	1.843 (1.456–3.641)	< 0.001	1.973 (1.596–3.974)	< 0.001	1.686 (1.284–2.973)	< 0.001
High level of CRP*High level of PCT		1.201 (1.093–1.839)	0.042	1.066 (0.861–2.415)	0.069	1.042 (0.892–2.241)	0.073

Model II: adjusting the fever days, hospital stay days, abnormal signs of lungs on the basis of Model I; Model III: adjusting IgA, IgG on the basis of Model II.

**Table 6:** Additive interaction of related indicators.

Variable		RERI (95% CI)^#^	AP (95% CI)^#^	S (95% CI)^#^
NNT-AS1	CRP	2.753 (0.363, 4.961)	0.415 (0.285, 0.952)	1.531 (1.276, 4.638)
NNT-AS1	PCT	1.164 (0.741, 1.475)	0.324 (0.175, 0.579)	1.186 (1.044, 2.617)
CRP	PCT	1.341 (− 0.729, 2.639)	0.207 (− 0.231, 0.472)	2.427 (0.565, 3.328)

^#^Adjusting the fever days, hospital stay days, abnormal signs of lungs, IgA, IgG.

## Discussion

Most studies believe that MP infection can stimulate the activation of T cells in the body, promote the high expressions of cytokines and inflammatory factors in the body, and aggravate the inflammatory response in the body, thus leading to RMPP^16^. Moreover, prolonged fever during the treatment of RMPP will also cause excessive inflammatory response in the body, causing the disease course to be prolonged^17,18^. Studies have shown that the clinical manifestations of RMPP and NRMPP are not significantly different except for different sensitivities to macrolides antibiotic treatment. The symptoms of RMPP and NRMPP are both primarily fever and respiratory symptoms such as cough, with RMPP patients having longer fever time and hospital stay, and being more likely to have extra-pulmonary symptoms^19,20^. The results of this study were similar to these findings. The fever time, length of hospital stay and the incidence of abnormal pulmonary signs in children with RMPP were all significantly longer or higher than those in children with NRMPP. Meanwhile, we also found the IgA and IgG levels in children with RMPP both higher than those in NRMPP children, which may be associated with the anti-infection mechanism of the immune system: immune cells and the complement system are activated under the stimulus of mycoplasma pneumoniae, therefore inducing IgA and IgG to prevent the invasive injury, neutralize endotoxin and alleviate the pathological damage^21,22^; in RMPP patients, due to the prolonged illness, the adaptive immunity is activated and the immune globulin secreted by the B cells is increased.

CRP is an important biomarker that can reflect the severity of pneumonia and the injury in extra-pulmonary tissues. PCT is also an indicator of inflammation, the elevation of which has been found in many critically ill patients, possibly due to the presence of high concentration of pro-inflammatory cytokines in severe patients that overwhelms the immune system and leads to the systemic immune syndrome^23^. In this study, CRP and PCT were significantly increased in the RMPP patients, consistent with the results of relevant studies^6,9^. Both CRP and PCT could be potential biomarkers for predicting the occurrence of RMPP.

As newly discovered LncRNA, NNT-AS1 can affect cell proliferation, metastasis and apoptosis^24^, and is abnormally expressed in malignant tumors in the digestive system and the female reproductive system^25,26^. Study by Wang et.al^14^ showed that NNT-AS1 was highly expressed in colorectal cancer patients, and was closely related to lymph node metastasis, vascular invasion and differentiation. In this study, we found that, for the first time, the serum LncRNA NNT-AS1 level in children with RMPP was significantly higher than that in NRMPP children, and that high expression of LncRNA NNT-AS1 was a risk factor for RMPP. In addition, LncRNA NNT-AS1, CRP and PCT had synergistic effects on RMPP. There were also positive additive interactions between LncRNA NNT-AS1 and CRP, and between LncRNA NNT-AS1 and PCT. Moreover, the diagnostic efficiency of combined diagnosis was higher than those of the three single diagnoses on RMPP. Therefore, it was suggested that children with abnormal expressions of LncRNA NNT-AS1, CRP and PCT should be included as key screening subjects for RMPP and these three biomarkers need be controlled within the normal range.

In summary, LncRNA NNT-AS1, CRP and PCT were significantly higher expressed in children with RMPP; LncRNA NNT-AS1 had synergistic effects with CRP and PCT, respectively, on the occurrence of RMPP. Serum levels of LncRNA NNT-AS1, CRP and PCT need to be controlled to reduce the occurrence of RMPP. In addition, the combined diagnosis of the three biomarkers could better predict the occurrence of RMPP than any of the single-factor diagnoses, which was of great significance for the clinical diagnosis and targeted treatment of RMPP, and laid a theoretical foundation for further research on the mechanism of RMPP.

## Materials and methods

### General information and sample collection

From June 2018 to June 2020, 150 hospitalized children diagnosed with MPP infection were selected from the respiratory department in the Fujian Maternal and Child Health Care Hospital as study subjects. MPP diagnostic criteria were^27^: (1) antibody against mycoplasma pneumoniae MP-Ab > 1:160, (2) positive serum mycoplasma pneumoniae specific IgM antibody, (3) MP-DNA copy number > 500 in pharyngeal swabs, sputum, alveolar lavage fluid or pleural effusion, (4) excluding the pulmonary infection caused by other pathogens such as virus and bacteria. In accordance with any of the (1) to (3) critetia plus criteria (4) can be confirmed as an MPP case.

Diagnostic criteria of RMPP were^3^ : if either of the following situations occurs in MPP patients treated with macrolide antibiotics for 7 days or above: (1) the condition of the child is not improved, such as aggravated clinical symptoms or continuous fever, (2) lung imaging shows aggravated developments of unilateral or bilateral large-lobe high-density lung consolidation, combined with or without pleural effusion, diffuse interstitial lung infiltration, and atresia.

Exclusion criteria :(1) patients receiving oral or intravenous glucocorticoids or 2 and more antibiotics within 3 days before admission, (2) patients had have or currently having chronic pulmonary diseases such as broncho-pulmonary dysplasia, bronchiectasis, recurrent respiratory infections, as well as patients with immune dysfunction, congenital heart disease, chronic nephritis, SLE, malnutrition, diabetes and other genetic metabolic diseases, (3) patients infected with other pathogens detected within 1 week after admission, (4) convalescent patients from pneumonia or mycoplasma pneumoniae or patients infected within 1 month before admission.

3 ml of venous blood sample was taken from the subjects on an empty stomach the next morning after hospitalized into a blood vessel premixed with EDTA, then centrifuged at 4 ℃ (1000×*g*, 10 min). The upper liquid was pipetted out carefully and transferred into a precooled EP tube, and was centrifuged at 4 ℃ (14,000×*g*, 15 min). The supernatant was then moved into an enzyme-free EP tube, and stored at 80 ℃ for further analyze.

### Real-time fluorescent quantitative PCR (qRT-PCR)

Total RNA was extracted from serum samples with TRIzol reagent (Invitrogen, USA). The total RNA purity was detected by ultraviolet spectrophotometer, the OD260/OD280 of the RNA samples that met the requirements of this study were located between 1.8 and 2.0. Total RNA was reverse transcribed into cDNA using the High-capacity cDNA Reverse Transcription Kit (Thermo Fisher, USA). The expressions of serum lncRNA NNT-AS1 and internal reference gene GAPDH were detected using qRT-PCR, and the reaction system was as follows: 10 μL of mix, 1 μL each of upstream and downstream primers, 2 μL of cDNA, and 6 μL of DEPC water. The PCR amplification conditions were as follows: 95 ℃ for 5 min, then 35 cycles of 95 ℃ for 30 s, 60 ℃ for 30 s, and 72 ℃ for 30 s. We used 2^−ΔΔCt^ to indicate the relative gene expression level. ΔΔCt = [(Ct_exp purpose gene_ − Ct_exp ref gene_) − (Ct_cnt purpose gene_ − Ct_cnt ref gene_)]^28^.lncRNA NNT-ASL forward primers: 5'-TGAAGTTTTCAGGGACACAT-3′, reverse primers: 5′-TTTAGACCTGTTTCTTTGTT-3′, GAPDH forward primers: 5′-CTCTTCCTTCCTTCCTTCCTTCCT-3′, reverse primers: 5′-AGCACTGTGTTGGCGTACAG-3'.

### Tests of laboratory indicators

The white blood cell (WBC) count, neutrophil count and platelet count were measured by ADVIA 2120I automatic hematology analyzer (Siemens, Germany). The IMMAGE 800 automatic specific protein analyzer (Beckman Coulter, USA) and matching reagent are used to detect IgA, IgG and IgM. The Monitor-100 automatic dynamic blood sedimentation analyzer (Vital company, Italy) was used for the detections of erythrocyte sedimentation rate (ESR). Serum D-Dimer level was detected by latex enhanced immunoturbidimetry and serum LDH level was detected by the rate method. CRP was measured by immune transmission turbidimetric method using Hitachi 7600 series automatic biochemical analyzer. PCT was tested by chemiluminescence method, using MAGLUMI 1000 automatic chemiluminescence instrument and supporting reagent (produced by Shenzhen New Product Biomedical Engineering Co., LTD.).

### Statistical method

Data were analyzed using SPSS (version 22.0). The normal distribution of quantitative data was described by mean ± standard deviation, and t-test was used for comparison between the two groups. Categorical data were tested using chi-square test. The correlations between lncRNA NNT-ASL, CRP and PCT were analyzed by Pearson correlation test.

The predictive abilities of the indicators were evaluated by the area under the Receiver Operating Characteristic Curve (ROC curve). Logistic regression crossover model was used to analyze the multiplying interactions of LncRNA NNT-ASL, CRP and PCT, and the EXCEL compiled by Andersson et al. was used to analyze the additive interactions^29,30^. The relative excess risk due to interaction (RERI), the attributable proportion due to interaction (AP), and the synergy Index (S) were used to evaluate the additive interactions. If there was additive interaction between any two factors, the confidence interval of RERI and AP should not include 0, and the trust interval of S should not include 1. P < 0.05 was considered statistically significant.

### Ethics statements

All methods were carried out in accordance with relevant guidelines and regulations. The experimental protocols were approved by Fujian Maternity and Child Ethics committee (2020KY112). And the informed consent was obtained from a parent and/or legal guardian.
